# Reliable quantification of ^18^F-GE-180 PET neuroinflammation studies using an individually scaled population-based input function or late tissue-to-blood ratio

**DOI:** 10.1007/s00259-020-04810-1

**Published:** 2020-04-23

**Authors:** Ralph Buchert, Meike Dirks, Christian Schütze, Florian Wilke, Martin Mamach, Ann-Katrin Wirries, Henning Pflugrad, Linda Hamann, Laura B.N. Langer, Christian Wetzel, Mario Lukacevic, Andras Polyak, Mariella Kessler, Carlotta Petrusch, Frank M. Bengel, Lilli Geworski, Rainer Rupprecht, Karin Weissenborn, Tobias L. Ross, Georg Berding

**Affiliations:** 1 Department of Diagnostic and Interventional Radiology and Nuclear Medicine, University Medical Center Hamburg-Eppendorf, Martinistr. 52, 20246 Hamburg, Germany; 2 Department of Neurology, Hannover Medical School, Hannover, Germany; 3 Department of Medical Physics and Radiation Protection, Hannover Medical School, Hannover, Germany; 4 Department of Nuclear Medicine, Hannover Medical School, Hannover, Germany; 5 Department of Psychiatry and Psychotherapy, University of Regensburg, Regensburg, Germany

**Keywords:** Translocator protein (TSPO), Flutriciclamide, GE-180, Kinetic analysis, Input function, Population-based

## Abstract

**Purpose:**

Tracer kinetic modeling of tissue time activity curves and the individual input function based on arterial blood sampling and metabolite correction is the gold standard for quantitative characterization of microglia activation by PET with the translocator protein (TSPO) ligand ^18^F-GE-180. This study tested simplified methods for quantification of ^18^F-GE-180 PET.

**Methods:**

Dynamic ^18^F-GE-180 PET with arterial blood sampling and metabolite correction was performed in five healthy volunteers and 20 liver-transplanted patients. Population-based input function templates were generated by averaging individual input functions normalized to the total area under the input function using a leave-one-out approach. Individual population-based input functions were obtained by scaling the input function template with the individual parent activity concentration of ^18^F-GE-180 in arterial plasma in a blood sample drawn at 27.5 min or by the individual administered tracer activity, respectively. The total ^18^F-GE-180 distribution volume (V_T_) was estimated in 12 regions-of-interest (ROIs) by the invasive Logan plot using the measured or the population-based input functions. Late ROI-to-whole-blood and ROI-to-cerebellum ratio were also computed.

**Results:**

Correlation with the reference V_T_ (with individually measured input function) was very high for V_T_ with the population-based input function scaled with the blood sample and for the ROI-to-whole-blood ratio (Pearson correlation coefficient = 0.989 ± 0.006 and 0.970 ± 0.005). The correlation was only moderate for V_T_ with the population-based input function scaled with tracer activity dose and for the ROI-to-cerebellum ratio (0.653 ± 0.074 and 0.384 ± 0.177). Reference V_T_, population-based V_T_ with scaling by the blood sample, and ROI-to-whole-blood ratio were sensitive to the TSPO gene polymorphism. Population-based V_T_ with scaling to the administered tracer activity and the ROI-to-cerebellum ratio failed to detect a polymorphism effect.

**Conclusion:**

These results support the use of a population-based input function scaled with a single blood sample or the ROI-to-whole-blood ratio at a late time point for simplified quantitative analysis of ^18^F-GE-180 PET.

**Electronic supplementary material:**

The online version of this article (10.1007/s00259-020-04810-1) contains supplementary material, which is available to authorized users.

## Introduction

There is increasing evidence that chronic neuroinflammation caused by cells of the innate neuroimmune system after activation by danger-associated molecular patterns such as misfolded proteins contributes to the pathogenesis of neurodegenerative diseases [1, 2]. Chronic pro-inflammatory reactions of the neuroimmune system most likely play a prominent role also in various other neurological and psychiatric diseases including stroke [3], multiple sclerosis [4], brain tumors [5], hepatic encephalopathy [6], and major depression [7].

Microglia is the major cell type of the neuroimmune system [1]. Its activation is associated with an increased expression of the translocator protein (TSPO), an 18-kDa, five transmembrane domain protein primarily located in the outer mitochondrial membrane and formerly known as peripheral benzodiazepine receptor [8–11]. Positron emission tomography (PET) imaging with radiolabeled TSPO ligands therefore is a promising modality for detection, quantitative characterization, and monitoring of neuroinflammation in vivo [12]. In patients with mild cognitive impairment or mild dementia of the Alzheimer type, for example, PET with the first-generation TSPO ligand [^11^C](R)-PK11195 [13] demonstrated increased tracer binding in the brain regions with the most prominent synaptic dysfunction/degeneration in Alzheimer’s disease, suggesting microglial activation at early clinical stages of the disease [14].

Limitations of [^11^C](R)-PK11195 include its rather low signal-to-background binding ratio in vivo [15] and the short 20 min physical half-life of the radioactive label. The latter restricts the use of [^11^C](R)-PK11195 to centers with cyclotron and radiochemistry on site. This prompted the development of novel TSPO PET ligands with improved pharmacokinetics and labeled with ^18^F (110 min half-life). Amongst these second- and third-generation TSPO PET ligands is the tricyclic indole compound (S)-N,N-diethyl-9-(2-[^18^F]fluoroethyl)-5-methoxy-2,3,4,9-tetrahydro-1H-carbazole-4-carboxamide (Flutriciclamide, ^18^F-GE-180) [16]. Superior pharmacokinetics (higher binding potential) compared to [^11^C](R)-PK11195 has been demonstrated in a lipopolysaccharide-induced rat model of acute neuroinflammation [17] and in a rat model of stroke [18]. ^18^F-GE-180 provided higher sensitivity to detect microglial activation and its changes under therapy in a mouse model of Alzheimer’s disease compared to the second-generation TSPO tracer ^18^F-PBR06 [19].

The standard method for quantitative characterization of ^18^F-GE-180 binding in humans is full tracer kinetic modeling of tissue time activity curves (TAC) measured by PET via acquisition of a sequence of image frames covering a total duration of at least 90 min starting with injection of ^18^F-GE-180 [20, 21]. The input function required for full kinetic modeling, that is, the time course of unmetabolized ^18^F-GE-180 in arterial plasma in brain capillaries, is derived from automatically and/or manually drawn arterial blood samples using high-performance liquid chromatography (HPLC) analysis to separate radioactive metabolites. Arterial blood sampling during the whole scan duration and HPLC processing of (≥ 6) discrete blood samples is not only burdensome for both patient and staff but it also restricts the use of ^18^F-GE-180 PET to centers with radiochemical facility.

Reference tissue methods that allow quantitative estimates of tracer binding by comparing the TAC in the region-of-interest (ROI) with the TAC in a reference tissue region free of the imaging target (TSPO) and, therefore, do not require the arterial input function, are widely used. Application of reference tissue methods in TSPO PET is limited by the lack of a brain region in which microglial activation can be ruled out a priori in all subjects under all conditions [20]. Supervised clustering of tissue TACs on the voxel level [22, 23], voxel-based statistical testing of a late standard uptake value (SUV) image of healthy controls versus a group of patients with the disease of interest [24], and crescent-shaped ROIs manually placed in brain regions with visually normal tracer uptake [25] have been proposed for the identification of an appropriate tissue reference region in TSPO PET. These methods have been proven useful for quantitative characterization of lesions with high microglial activation. They might be limited if there is no valid reference region due to possible widespread global neuroinflammation.

Population-based input functions for tracer kinetic modeling have been successfully used for quantitative analysis of brain PET with FDG [26–29] and a variety of other tracers [30–35]. The aim of the present study was to evaluate methods for quantitative analysis of ^18^F-GE-180 PET using population-based blood curves that either require only a single late blood sample or no blood at all. The tissue-to-whole-blood ratio at a late time point was also tested. The latter does not require dynamic PET imaging (only a late static uptake image) and has been shown to be an excellent surrogate of quantitative parameters from modeling of dynamic PET data for other tracers [36, 37].

## Materials and methods

### Subjects

The study included a total of 25 subjects (age 59.4 ± 9.4 years, range 37–77 years, 7 females), 20 patients after liver transplantation and 5 healthy subjects from an ongoing prospective study on the effect of immunosuppression on microglial activity in liver-transplanted patients. Nine of the 20 liver-transplanted patients were on standard dose immunosuppression with the calcineurin inhibitors (CNI) tacrolimus or ciclosporin with or without combination with other immunosuppressants such as mycophenolat mofetil. Nine liver-transplanted patients were on reduced CNI dose. The remaining two liver-transplanted patients were on immunosuppressive therapy free of CNI. All patients after liver transplantation included in this study had restored liver function.

### DNA extraction and polymorphism genotyping

The binding of most (if not all) TSPO ligands is affected by a single nucleotide polymorphism in the TSPO gene (SNP rs6971) leading to an Ala147Thr amino-acid substitution which affects affinity of the TSPO for binding of the PET ligands. This results in systematic inter-subject variability of TSPO ligand kinetics depending on the genotype (high-affinity binder, HAB, or low-affinity binder, LAB, or mixed affinity binder, MAB) [38].

Genotyping was performed as described previously [25]. In brief, genomic DNA was extracted from 4 mL of whole blood with QIAamp DNA blood maxi kit (Qiagen, Hilden, Germany) according to the manufacturer’s protocol. DNA quality was assessed utilizing optical absorbance and gel electrophoresis. Exon 4 of TSPO gene, as well as exon/intron junctions, were PCR amplified and sequenced using the Sanger method with the following primers: ex4-F-AGTTGGGCAGTGGGACAG and ex4-R-GCAGATCCTGCAGAGACGA. Sequencing data were analyzed using SnapGene software (GSL Biotech; available at snapgene.com).

Fourteen subjects (11 patients/3 healthy control subjects) were HAB, 8 subjects (7/1) were MAB, and 3 subjects (2/1) were LAB (Table 1). HAB subjects were slightly older than MAB subjects, the difference just barely missed statistical significance (61.6 ± 9.3 versus 54.0 ± 9.0 years, two-sided *t* test *p* = 0.077). HAB subjects and MAB subjects did not differ with respect to sex (36 versus 25% females, chi-square *p* = 0.604).

**Table 1:** Number of subjects (mean age ± standard deviation, age range) according to group and TSPO genotype

	LAB	MAB	HAB
Healthy subjects	1 (63)	1 (50)	3 (63 ± 11, 50–71)
Patients after liver transplantation	2 (64 ± 10, 57–71)	7 (55 ± 9, 37–68)	11 (61 ± 9, 44–77)

### Synthesis of ^18^F-GE-180

^18^F-GE-180 was produced in a GMP-compliant synthesis using a single use disposable cassette (FASTlab PET GE-180 cassette, GE Healthcare, UK) on an automated synthesizer system (FASTlab™, GE Healthcare, UK) and the corresponding *S*-enatiomeric pure mesylate precursor (3.5 mg, GMP grade) [39]. [^18^F]fluoride was obtained from a 11-MeV cyclotron (Eclipse HP, Siemens, Knoxville, USA) using the ^18^O(p,n)^18^F nuclear reaction on enriched (97–98%) [^18^O]water, and directly transferred to the radiosynthesizer. After 45 min automated synthesis, the product ^18^F-GE-180 was obtained as the pure *S*-enatiomer (*S*)-*N,N*-diethyl-9-(2-[^18^F]fluoroethyl)-5-methoxy-2,3,4,9-tetrahydro-*1H*-carbazole-4-carboxamide [40] in a sterile buffer solution (35–37 ml). The radiochemical yield was 42% ± 5% with radiochemical purity of ≥ 97% and specific activity of 517 ± 54 GBq/μmol. Quality control tests were performed according to GMP and the EU pharmacopoeia. All batches met the required acceptance criteria and were released for human administration.

### PET imaging

PET imaging was performed with a Biograph mCT (Siemens, Erlangen, Germany). A list mode emission scan of 90 min duration was started simultaneously with the intravenous injection of 178 ± 6 MBq (range 165–195 MBq) ^18^F-GE-180 over 10 s. Mean specific dose was 2.11 ± 0.35 MBq/kg bodyweight (range 1.62–3.11 MBq/kg). Specific dose did not differ between TSPO Ala147Thr genotypes (2.17 ± 0.39 MBq/kg, 2.00 ± 0.35 MBq/kg, and 2.10 ± 0.15 MBq/kg in HAB, MAB, and LAB, respectively, univariate analysis of variance *p* = 0.586).

PET emission data were reconstructed by filtered backprojection into a sequence of 26 image frames according to the following protocol: 8 × 15 s, 3 × 60 s, 5 × 120 s, 5 × 300 s, 5 × 600 s. Voxel size was 1.57 × 1.57 × 2.00 mm^3^ (matrix size 200 × 200, zoom factor 2.6). A low-dose CT (100 kV, 28 eff. mAs) acquired immediately prior to the PET emission scan was used for attenuation correction. Correction for random coincidences, scatter, and radioactive decay was applied as implemented in the system software. Reconstructed images were post-filtered with an isotropic Gaussian kernel with 5 mm full-width-at-half-maximum (FWHM) [20, 21].

### Whole-blood time activity curve and input function

The time course of radioactivity concentration in arterial whole blood during the first 15 min after start of tracer injection was measured with an automatic blood sampling device (Veenstra PBS-101, Veenstra Instruments, The Netherlands [41]). During the first 4 min, blood was drawn from the radial artery at a rate of 5 ml/min and activity concentration was measured 2 times per second. During the subsequent 11 min, blood was drawn at a rate of 2.5 ml/min and measured once every 3 s. In addition, 12 arterial blood samples were drawn manually at the midtime of the PET imaging frames 11, 14, and 17–26, that is, at 4.5, 10, 17.5, 22.5, 27.5 32.5, 37.5, 45, 55, 65, 75, and 85 min after the start of tracer injection. Whole-blood activity concentration in the manual blood samples was measured with a well-counter (Wizard 2470, Perkin Elmer Inc., Waltham, MA, USA) cross-calibrated to the PET scanner. The automatic blood sampler was calibrated separately for each subject by comparing the activity concentration of the manual blood samples at 4.5 and 10 min with the blood sampler measurements at these time points.

The whole-blood time activity curve measured with the automatic blood sampler was corrected for delay and dispersion relative to the whole-blood time activity curve in the brain as described in the online supplementary (subsection “Correction for delay and dispersion”).

The arterial blood samples manually drawn at 4.5, 10, 17.5, 32.5, 65, and 85 min were used to determine the plasma-to-whole-blood radioactivity concentration ratio and the (parent) fraction of unmetabolized ^18^F-GE-180 in arterial plasma at these time points. One milliliter of arterial blood was separated into plasma and blood cell fraction by centrifugation (3′000 rcf) at 4 °C for 15 min. Two hundred fifty microliters of the plasma fraction was mixed with 250 μl ice cold methanol for degradation of proteins. Serum and proteins were separated by centrifugation (10′000 rcf) at 4 °C for 5 min. The serum fraction was transferred and cleared by a second centrifugation (10′000 rcf) at 4 °C for 10 min. The upper layer of the serum was transferred into a sample vial. The latter was measured by HPLC (Merck-Hitachi LaChrom-HPLC-system equipped with a Raytest GABI radiodetector) using a Phenomenex Chromolith RP-18e, 100 × 4.6 mm, column, and an isocratic eluent of methanol/water (60:40) at 1 ml flow. An injection volume of 200 μl was used, and fractions of 90 s (1.5 ml) were collected over 0–12 min and measured separately in the well-counter. Metabolites occurred at 2–7 min, parent compound at 8–10 min. Individual plasma-to-whole-blood time curves were fitted by a constant, because the data did not suggest another, more complex model, in line with previous findings [20, 21]. Individual parent fraction time curves were fitted by a single exponential plus constant model as described by Feeney and co-workers [21]. More precisely, individual parent fraction time curves were fitted by 1 - a * [1 - exp (−μ*t)], where a and μ are the free parameters to be optimized and t is the sampling time. Fan and colleagues used a 2-exponential linear model to describe the time course of the parent fraction of ^18^F-GE-180 in plasma [20]. In our data, this slightly more complex model did not improve the quality of the fit compared to the single exponential plus constant model.

The individual input function for tracer kinetic modeling was obtained as follows: input function = plasma-to-whole-blood ratio * parent fraction * whole-blood time activity curve.

### Magnetic resonance imaging

High-resolution T1-weighted magnetization prepared rapid acquisition gradient echo (MP-RAGE) magnetic resonance images (MRI) were obtained using a 3-T Verio MRI system (Siemens, Erlangen, Germany; voxel size 1.0 × 1.0 × 1.0 mm^3^, echo time 2.93 ms, repetition time 1900 ms, inversion time 900 ms, flip angle 9.0°).

### Image pre-processing and tissue time activity curves

Frame-wise correction of head motion during the PET emission scan was performed using the Realign-tool of the statistical parametric mapping software (version SPM12, https://www.fil.ion.ucl.ac.uk/spm/). Frames 7 to 26 (90 s–90 min) were included in the realignment as they provided sufficient anatomical information for reliable estimation of the rigid body transformation for realignment. The last frame was used as reference. The realignment transformation of frame 7 was also applied to frames 1–6.

The rigid body transformation to map the dynamic PET image sequence to the subject’s MRI was estimated using the Coregister-tool of SPM12 with the individual MRI as target image. The static PET image obtained by integrating the motion-corrected frames 7 to 26 was used as source image.

The individual MRI was stereotactically normalized into the anatomical space of the Montreal Neurological Institute (MNI) using the Normalize-tool of SPM12 [42]. The patient’s PET image sequence was resliced to MNI space in a single step that combined the coregister transformation to individual MRI space and stereotactical normalization from individual MRI space to MNI space.

TACs of frontal cortex, parietal cortex, temporal cortex (without mesial temporal cortex), occipital cortex, cerebellum, insula, cingulate cortex, mesial temporal cortex, precuneus, striatum, thalamus, and the superior longitudinal fasciculus were obtained by applying binary masks of these regions predefined in MNI (s. subsection “Brain regions of interest” in the online supplementary).

### Reference quantification method

The invasive graphical Logan method [43] implemented in a custom-made MATLAB script was used to estimate the regional total distribution volume V_T_ (unit = mL blood/cm^3^ tissue) from the regional tissue TACs and the individual arterial input function. The operational equation of the invasive graphical Logan method is [43].

1 $$ {\int}_o^t\mathrm{TAC}(s) ds/\mathrm{TAC}(t)={V}_T\ {\int}_0^t{C}_P(s)\mathrm{ds}/\mathrm{TAC}(t)+\mathrm{const},\kern0.5em t\ge {t}^{\ast }, $$

where TAC is the time activity curve of the tissue ROI, *C*_*P*_ is the input function, and *t*^∗^ is the time at which the plot of ${\int}_o^t\mathrm{TAC}(s)\mathrm{ds}/\mathrm{TAC}(t)$ versus ${\int}_0^t{C}_P(s)\mathrm{ds}/\mathrm{TAC}(t)$ reaches linearity. According to its operational equation, the invasive graphical Logan method involves the area under the input function from time *t* = 0 to times *t* ≥ *t*^∗^ only. It therefore might be less sensitive than nonlinear methods (that estimate V_T_ by combining individual rate constants) to deviations of the population-based input function from the actual input function at early times (< *t*^∗^) at which a population-based input function might not accurately reproduce the rapid changes of individual input functions [33, 34]. This is the rationale for using the invasive graphical Logan method with population-based input functions. Fan and co-workers reported high correlation between V_T_ estimates obtained by the invasive Logan plot and V_T_ estimates obtained by the reversible 2-tissue compartment model in both HAB subjects (Pearson’s correlation coefficient R in frontal, temporal, parietal, occipital lobe, and hippocampus ≥ 0.96) and MAB subjects (R ≥ 0.85) [20].

The regional tissue TACs were corrected for fractional blood volume using the individual whole-blood TAC and assuming a fixed fractional blood volume of 5% [21]. Correction of tissue TACs for fractional blood volume is particularly relevant in ^18^F-GE-180 PET, because in healthy brain tissue about 20% of the PET signal is from fractional blood volume even at late time points [21]. The start of the linear fit was fixed at frame 20 so that the linear fit included the data from 30 to 90 min after tracer injection, as proposed by Zanotti-Fregonara and co-workers [44]. The fit range appropriately covered the linear part of the invasive Logan plot in all ROIs in all subjects (according to visual inspection). Some previous studies used the maximum admissible error criterion [45] to select the time start point t* of the linear fit in graphical tracer kinetic modeling. However, this criterion tends to cause outliers [46], in particular when the same fixed maximum error is used for all ROIs [45]. In the present study, a fixed start point t* was used in order to avoid outliers. Conventional linear regression was used to fit a straight line to the Logan plot. No effort was made to reduce noise-associated bias [47].

The invasive graphical Logan method with measured blood curves was used as reference method in this study.

### Population-based input functions

Population-based input function approaches involve two steps [26, 32]. The first step is to generate an input function template (*IFT*) representing the typical shape of the input function across subjects. The second step is to generate population-based input functions (*PBIF*) for individual subjects from the input function template. Both steps involve scaling. In order to avoid overly optimistic performance estimates, a leave-one-out approach was used for the generation of the input function template, that is, subject *i* was excluded from the generation of the input function template *IFT*_*i*_ that later was used to generate the population-based input function for subject *i* [31, 33, 48]. More precisely

2 $$ {\mathrm{IFT}}_i=\frac{1}{n-1}{\sum}_{\begin{array}{c}j=1\\ {}j\ne i\end{array}}^n{\mathrm{MIF}}_j/{\mathrm{SFIF}}_j^{\left(\mathrm{IFT}\right)} $$

where *n* (= 25) is the total number of subjects, *MIF*_*j*_ is the measured input function of subject *j*, and the sum on the right hand side excludes subject *i*. Normalization of the measured input function *MIF*_*j*_ to the scale factor ${SFIF}_j^{(IFT)}$ is intended to reduce inter-subject variability (of the amplitude) of the input functions. Scaling to the total area under the input function was used for the generation of the input function template [26, 28, 32], that is

3 $$ {\mathrm{SFIF}}_j^{\left(\mathrm{IFT}\right)}={\int}_0^{90\ \mathit{\min}}{\mathrm{MIF}}_j(t)\mathrm{dt} $$

Prior to averaging the scaled individual input functions according to the right hand side of formula (2), each input function was shifted in time (by a few seconds) to achieve a common position of the peak across all scaled input functions to be averaged. The average peak position of the unshifted input functions was used as common peak position. In addition, each scaled input function was interpolated to a common time grid (every second during the first 5 min, followed by every 5 s until 15 min post injection, followed by the midtimes of the PET frames, that is, 17.5, 22.5, 27.5, 32.5, 37.5, 45, 55, 65, 75, and 85 min post injection).

The population-based input function *PBIF*_*i*_ of subject *i* was computed as

4 $$ {\mathrm{PBIF}}_i={\mathrm{SFIF}}_i^{(s)}\left({\sum}_{\begin{array}{c}k=1\\ {}k\ne i\end{array}}^n{\mathrm{SFIF}}_k^{\left(\mathrm{IFT}\right)}/{\sum}_{\begin{array}{c}k=1\\ {}k\ne i\end{array}}^n{\mathrm{SFIF}}_k^{(s)}\right)\ {\mathrm{IFT}}_i $$

where ${SFIF}_i^{(s)}$ is a simplified individual scale factor for subject *i*. The following simplified scaling methods were tested for computation of the population-based input function according to formula (4):

population-based method 1

5a $$ \left(\mathrm{PB}1\right):{\mathrm{SFIF}}_i^{(1)}={\mathrm{MIF}}_i\left(t={T}_0\right) $$

5b $$ \mathrm{PB}2:{\mathrm{SFIF}}_i^{(2)}=\mathrm{mean}\ \mathrm{plasma}\ \mathrm{to}\ \mathrm{whole}\ \mathrm{blood}\ \mathrm{ratio}\left({T}_0\right)\ast \mathrm{mean}\ \mathrm{parent}\ \mathrm{fraction}\left({T}_0\right)\ast {\mathrm{WB}}_i\left({T}_0\right) $$

5c $$ \mathrm{PB}3\ \left(\mathrm{SUV}-\mathrm{like}\right):{\mathrm{SFIF}}_i^{(3)}=\mathrm{activity}\ \left(\mathrm{MBq}\right)\ \mathrm{per}\ \mathrm{kg}\ \mathrm{body}\ \mathrm{weight}\ \mathrm{administered}\ \mathrm{to}\ \mathrm{subject}\ i $$

Here, *WB*_*i*_(*T*_0_) is the measured whole-blood activity concentration at time *T*_0_ in subject *i*. The time *T*_0_ of the single blood sample for scaling was selected according to the highest correlation between the activity concentration of unmetabolized ^18^F-GE-180 in arterial plasma and the total area under the measured input function, that is, highest correlation of ${SFIF}_i^{(1)}$ with the optimal scaling factor ${SFIF}_i^{(IFT)}$ across all subjects.

In order to account for fractional blood volume also with each of the three population-based methods, a whole-blood TAC template was obtained analogous to formula (2) and scaling to the area under the individual whole-blood TAC. The scale factors for generation of the population-based whole-blood TACs analogous to formulas (5a) were

6a $$ \mathrm{PB}1:{\mathrm{SFWB}}_i^{(1)}={\mathrm{WB}}_i\left(t={T}_0\right) $$

6b $$ \mathrm{PB}2:{\mathrm{SFWB}}_i^{(2)}={\mathrm{WB}}_i\left(t={T}_0\right) $$

6c $$ \mathrm{PB}3:{\mathrm{SFWB}}_i^{(3)}=\mathrm{activity}\ \left(\mathrm{MBq}\right)\ \mathrm{per}\ \mathrm{kg}\ \mathrm{body}\ \mathrm{weight}\ \mathrm{administered}\ \mathrm{to}\ \mathrm{subject}\ i $$

Each of the 3 different population-based methods was used with the invasive Logan plot to estimate V_T_ for each subject and each ROI. In addition, the ROI-to-whole-blood ratio (= ROI activity concentration in the last frame/whole-blood activity concentration in the 85 min blood sample) and the ROI-to-tissue-reference ratio in a sum image of the last 3 frames (60–90 min) was computed for each subject and each ROI. The cerebellum was used as pseudo-reference region, because it is amongst the brain regions with least altered ^18^F-GE-180 uptake in multiple sclerosis [24]. Furthermore, it has been demonstrated that the ROI-to-cerebellum ratio of the TSPO ligand [^11^C]PBR28 can serve as surrogate of V_T_ to detect increased TSPO availability in Alzheimer’s disease [49]. Table 2 gives a condensed overview of data required for the different simplified procedures.

**Table 2:** Requirements for quantification of ^18^F-GE-180 PET using the simplified methods. In addition, the table gives the Pearson correlation coefficient of the simplified quantitative parameter with the reference Logan V_T_ estimated with individual input function and whole-blood TAC (mean over all ROIs), and size of the TSPO polymorphism effect (HAB versus MAB) on the different quantitative parameters

Quantification method	Dynamic PET imaging (≥ 90 min)	Single late blood value	HPLC analysis	Correlation of the outcome measure with reference V_T_	Partial effect size η^2^ of the genotype effect
Population-based Logan plot 1 (PB1)	Required	Required	Required	0.989 ± 0.006	0.200
Population-based Logan plot 2 (PB2)	Required	Required	Not required	0.973 ± 0.007	0.179
Population-based Logan plot 3 (PB3)	Required	Not required	Not required	0.653 ± 0.074	0.010
ROI-to-whole-blood ratio	Not required	Required	Not required	0.970 ± 0.005	0.220
ROI-to-cerebellum ratio	Not required	Not required	Not required	0.384 ± 0.177	0.001

### Statistical analyses

Pearson’s correlation analysis (over the 25 subjects) was used to test the association of the population-based V_T_ values or the ROI-to-whole-blood or ROI-to-cerebellum ratio with the reference V_T_ values, separately for each ROI.

In addition, all quantitative parameters of regional ^18^F-GE180 binding (V_T_ values and ratios) were compared between MAB and HAB subjects independent of the group (liver-transplanted patient or healthy control).

## Results

Delay and dispersion time constant of the measured whole-blood TAC relative to the image-derived whole-blood TAC in the brain were 12.8 ± 3.7 s and 3.5 ± 1.8 s respectively (mean over all 25 subjects). Mean plasma-to-whole-blood ratio was 1.59 ± 0.10. The parent fraction of ^18^F-GE-180 in arterial plasma was 0.94 ± 0.02, 0.91 ± 0.04, 0.89 ± 0.06, 0.85 ± 0.06, 0.84 ± 0.05, and 0.80 ± 0.08 at 4.5, 10, 17.5, 32.5, 65, and 85 min after intravenous injection. The parameters a and μ of the exponential plus constant model used to fit the time course of the parent fraction were 0.23 ± 0.17 and 0.072 ± 0.061 min^−1^, respectively. None of the parameters listed so far differed between the 14 HAB subjects and the 8 MAB subjects except the parent fraction at 10 min, which was slightly lower in HAB subjects compared to MAB subjects (0.90 ± 0.03 versus 0.93 ± 0.04, t-test *p* = 0.031). The effect did not survive correction for multiple testing.

**Fig. 1:**
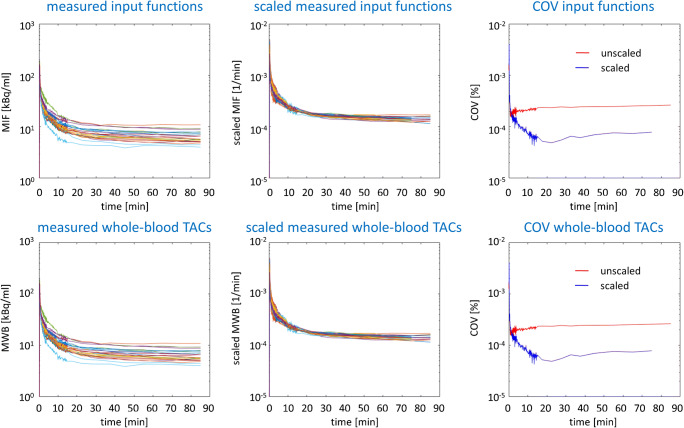
The top row shows the measured input functions (MIF) before (left) and after (middle) scaling to the area under the individual input function. The bottom row shows the measured whole-blood TACs (MWB) before (left) and after (middle) scaling to the area under the individual whole-blood TAC. The right column shows the time course of the coefficient of variance (COV) over all subjects with and without scaling for the input function (top) and for the whole-blood TAC (bottom). The figure includes the input functions and the whole-blood TACs from all 25 subjects. For the generation of population-based input functions and whole-blood TACs, a leave-one-out approach was used

Figure 1 shows arterial input functions and the arterial whole-blood TACs of all subjects as measured and after scaling to the total area under the individual curve. The time course of the coefficient of variance shows the reduction of inter-subject variability of input functions and whole-blood TACs by the scaling quantitatively (Fig. 1).

**Fig. 2:**
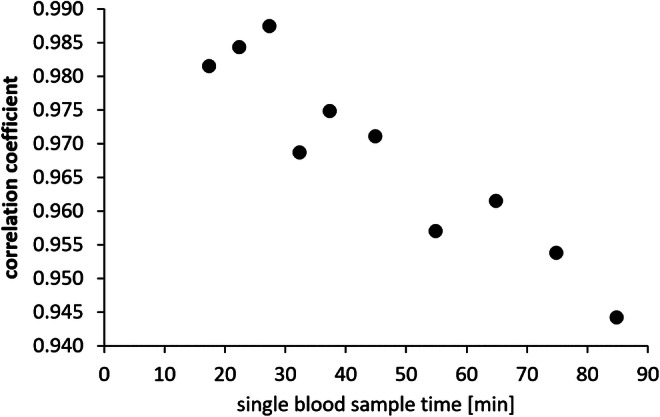
Pearson coefficient of the correlation between the activity concentration of unmetabolized ^18^F-GE-180 in arterial plasma (input function) and the total area under the measured input function for the different time points of manual blood sampling

The blood sample at *T*_0_ = 27.5 min showed the highest correlation between the activity concentration of unmetabolized ^18^F-GE-180 in arterial plasma and the total area under the measured input function (Fig. 2). Thus, the blood sample at *T*_0_ = 27.5 min was used for the population-based methods PB1 and PB2.

**Fig. 3:**
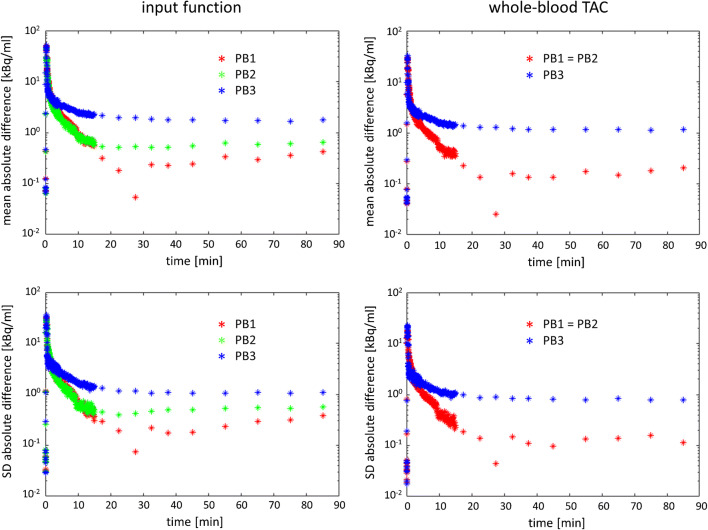
Mean (top row) and standard deviation (SD, bottom row) of the absolute difference between the population-based input function and the measured input function (left column) and between the population-based whole-blood TAC and the measured whole-blood TAC (right column). Mean and standard deviation of the absolute difference were computed over all 25 subjects included in this study. Population-based input functions and population-based whole-blood TACs were obtained using a leave-one-out approach. The different simplified scaling methods PB1, PB2, and PB3 are indicated by different colors. For comparison, the mean measured activity concentration of unmetabolized ^18^F-GE-180 in arterial plasma (input function) at t = 85 min was 5.1 kBq/ml (mean absolute difference between the population-based input function according to PB1 and the measured input function at 85 min = 0.42 ± 0.39 kBq/ml, that is, below 10% of the mean measured input function). The local minimum of mean and standard deviation at t = 27.5 min for scaling method PB1 is explained by the fact the blood sample at this time point was used for scaling

Mean and standard deviation of the absolute difference between the population-based and the measured input function over all 25 subjects are shown in Fig. 3. Both mean and standard deviation of the absolute difference show a peak during the first minute. This is explained by the fact that the population-based input function does not well describe the early phase of the true individual input function due to remaining inter-subject variability of the position, the height, and the width of the input function peak despite the use of a standardized injection protocol. This effect can be reduced by shifting the population-based input function such that its peak matches the peak of a whole-blood curve in the brain derived from the PET data [46]. However, the impact on the time integral of the input function at late time points (included in the linear fit of the invasive Logan plot) is negligible. As a consequence, the impact on V_T_ estimates is negligible, too. Individual time shift of population-based input functions was therefore not performed in this study. Mean and standard deviation of the absolute difference between the population-based input function and the measured input function reached a plateau at about 20 min post injection (Fig. 3). The plateau was lowest for the simplified scaling method PB1 and highest for the simplified scaling method PB3. The plateau for simplified scaling method PB2 was in-between. This suggests PB1 to be the best simplified scaling method, followed by PB2. Mean and standard deviation of the absolute difference between the population-based and the measured whole-blood TAC confirmed these findings (Fig. 3).

**Fig. 4:**
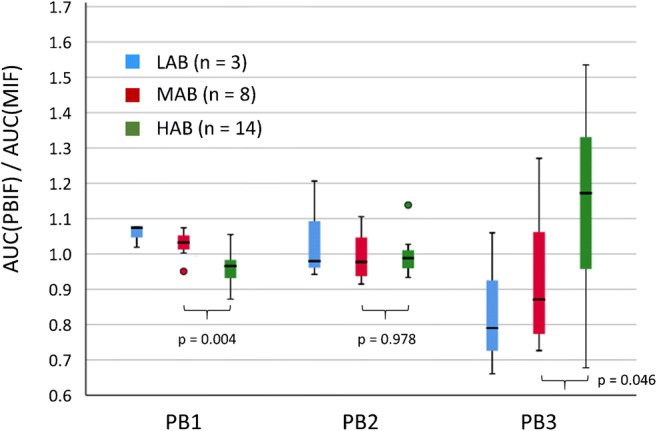
Box-and-whiskers plot of the ratio of the area (AUC) under the population-based input function (PBIF) to the AUC of the measured input function (MIF) for the different scaling methods PB1, PB2, and PB3. The colors indicate the TSPO genotype

The mean ratio of the area under the population-based input function relative to the area under the measured input function is shown in Fig. 4. The mean ratio over all subjects was close to 1 for all three scaling methods, but inter-subject variability was larger for PB3 compared to PB1 and PB2. Inter-subject variability of the ratio was smallest for PB1, providing further support for PB1 as the best amongst the tested simplified scaling methods. However, there was a small but statistically significant effect of the TSPO genotype with PB1: on average, there was an underestimation of the area under the input function in HAB subjects and an overestimation in MAB subjects (mean ratio with PB1 = 0.960 ± 0.050 and 1.027 ± 0.039 in HAB and MAB, respectively; *p* = 0.004). When the group (patients after liver transplantation versus healthy subjects) was taken into account, the genotype effect remained almost significant (*p* = 0.058) whereas the group had no effect (*p* = 0.925, univariate analysis of variance with the ratio as dependent variable and TSPO genotype, MAB or HAB, and group as fixed factors).

**Fig. 5:**
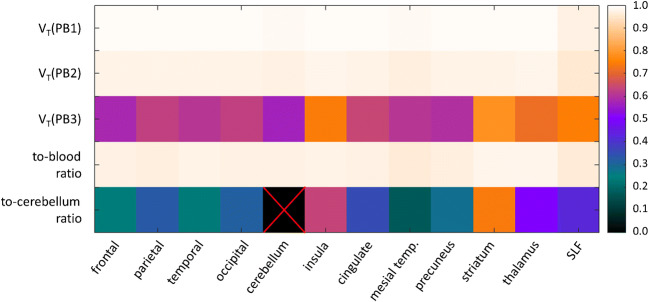
Heat map of the Pearson coefficient of the correlation of the total distribution volume V_T_ estimated with the different population-based methods (PB1–3) or the to-whole-blood ratio or the to-cerebellum ratio with the reference Logan V_T_ for all considered brain regions. It should be noted that the ROI-to-cerebellum ratio is a measure of specific binding relative to nondisplaceable tracer and therefore should approximate the nondisplaceable binding potential not V_T_ (whole tissue uptake relative to plasma input). Thus, inter-subject variability of nondisplaceable tracer binding affects the correlation of the ROI-to-cerebellum ratio with the reference Logan V_T_ (in addition to limitations of the ROI-to-cerebellum ratio to estimate the nondisplaceable binding potential)

Figure 5 shows the heat map of the Pearson coefficient for the correlation of the different simplified regional V_T_ estimates or the late concentration ratios with the regional reference V_T_ (estimated by the Logan plot with the measured input function). The correlation coefficient with the regional reference V_T_ was (i) 0.989 ± 0.006 (mean over all ROIs, range 0.971–0.992; all *p* < 10^−10^) for V_T_ estimated with the population-based method PB1, (ii) 0.973 ± 0.007 (0.955–0.980; all p < 10^−10^) for V_T_ estimated with the population-based method PB2, (iii) 0.653 ± 0.074 (0.570–0.782; all *p* < 0.003) for V_T_ estimated with the population-based method PB3, (iv) 0.970 ± 0.005 (0.960–0.978; all p < 10^−10^) for the regional to-whole-blood ratio, and (v) 0.384 ± 0.177 (0.174–0.741; in 4 of 11 ROIs *p* < 0.05) for the regional to-cerebellum ratio.

**Fig. 6:**
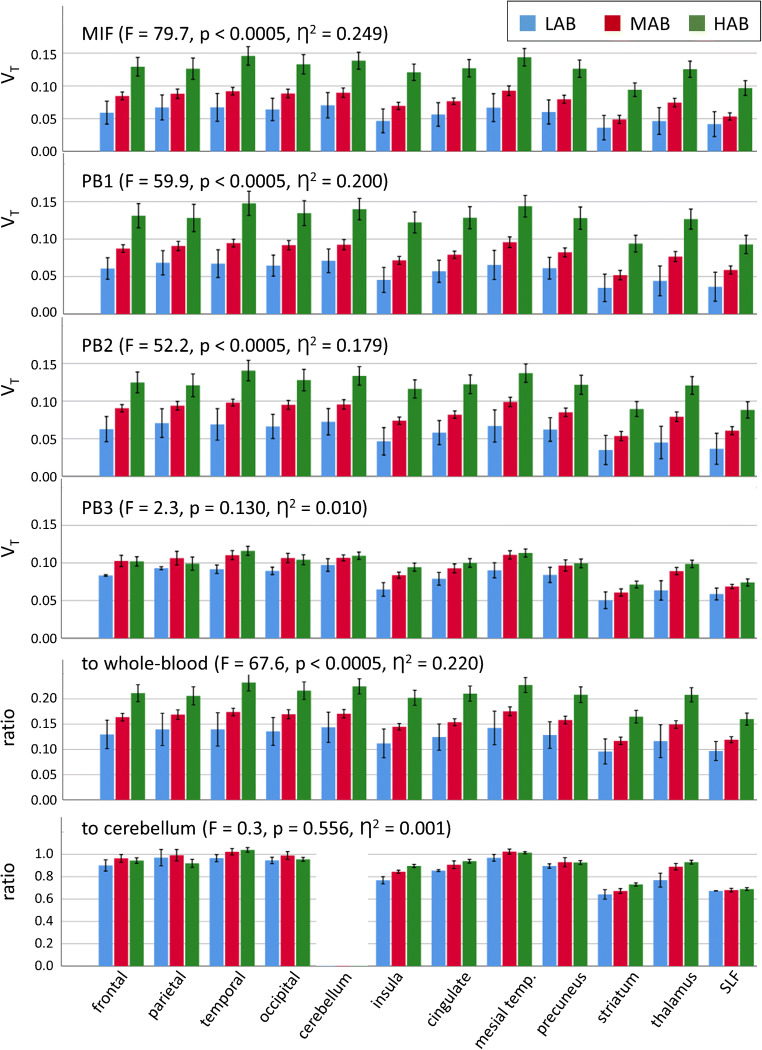
Comparison of the mean regional total distribution volume V_T_ (mL/cm^3^) between ROIs and TSPO genotypes (low-affinity binders LAB, mixed affinity binders MAB and high-affinity binders HAB). The error bars represent the standard error of the mean. The regional V_T_ was estimated by the graphical invasive Logan plot with measured input function (MIF) and measured whole-blood TAC (top row), or with population-based blood curves according to methods PB1 (2nd row), PB2 (3rd row), and PB3 (4th row). The ROI-to-whole-blood (5th row) and ROI-to-cerebellum ratio (bottom row) are also shown. F-statistic, *p* value, and partial effect size η^2^ of the polymorphism effect are from univariate analysis of variance with V_T_ or ROI-to-whole-blood ratio or ROI-to-cerebellum ratio as dependent variable and polymorphism (MAB or HAB) and region-of-interest as fixed factors (the LAB subjects were excluded from the analysis of variance because of the small number of LAB subjects in this study, *n* = 3)

The comparison of regional V_T_ between ROIs and TSPO genotypes is shown in Fig. 6. The regional V_T_ was significantly larger in HAB subjects than in MAB subjects when it was estimated by the graphical invasive Logan plot with measured blood curves or with population-based blood curves according to PB1 or PB2, or by the ROI-to-whole-blood ratio (univariate analysis of variance with TSPO polymorphism, HAB or MAB, and ROI as fixed factors: all *p* < 0.0005). There was no genotype * ROI interaction (*p* = 1.000). The mean ratio of reference V_T_ in HAB subjects relative to reference V_T_ in MAB subjects across all ROIs was 1.63 ± 0.14 (range 1.43–1.93). The V_T_ based on population-based method PB3 did not show a TSPO polymorphism effect (*p* = 0.130) nor did the ROI-to-cerebellum ratio (*p* = 0.556). The partial size η^2^ of the TSPO polymorphism effect (HAB versus MAB) on the different V_T_ estimates is given in Table 2.

## Discussion

The main requirement for population-based input function approaches to be suitable is that individual input functions show similar shape. In this case, individual input functions mainly differ in amplitude which can be easily accounted for by an individual scale factor. Input functions and whole-blood TACs of ^18^F-GE-180 fulfill this requirement to good approximation (Fig. 1), although there was an effect of the TSPO genotype: on average, there was an underestimation of the area under the input function in HAB subjects (about 4%) and an overestimation in MAB subjects (about 3%) by the population-based method PB1. The rather mild allelic sensitivity of the PB1 input function quality might be explained by the fact that scaling with the individual 27.5 min blood sample accounts for potential allelic differences of tracer metabolism and biodistribution at least partially (in contrast to SUV-like scaling) [34].

The primary finding of this study was the very strong correlation of the Logan V_T_ estimated with population-based blood curves scaled by a single blood sample (PB1 and PB2) as well as the ROI-to-whole-blood ratio at a late time point with the reference V_T_ across all brain regions (Table 2, Fig. 5). This suggests that these parameters are useful surrogates of the Logan reference V_T_ computed with the full arterial input function in brain PET with ^18^F-GE-180. Similar results have been reported by Mabrouk and co-workers who demonstrated the feasibility of TSPO quantification with [^18^F]FEPPA using a input function template scaled with a single blood sample [46]. The “costs” (including burden for the patient and the staff as well as financial costs) of parameter estimation are clearly lowest for the ROI-to-whole-blood ratio at a late time point (Table 2). Thus, the ROI-to-whole-blood ratio might be a useful compromise between validity as TSPO marker and costs. This is analogous to PET with [^18^F]fluorodeoxyglucose (FDG), where the (scan-time corrected) late lesion-to-blood uptake ratio shows excellent correlation with the FDG metabolic rate constant estimated by graphical analysis, better than the widely used standard uptake value (SUV) [36, 37].

The performance of population-based input functions might be improved by using two or more blood samples for individual scaling of the input function template [31, 33, 34, 46]. However, regional V_T_ estimates obtained with the population-based method PB1 using a single blood sample at 27.5 min were strongly correlated with the regional reference V_T_ in this study (mean Pearson coefficient over all ROIs = 0.989 ± 0.006, range 0.971–0.992; all *p* < 10^−10^). The use of additional blood samples cannot provide large improvement in this case and therefore was not tested here.

Some previous studies employed the same scaling method for the generation of the input function template from “old” subjects (with full blood sampling) as for the generation of individual population-based input functions from the input function template for “new” subjects [31, 32, 35]. In the present study, two different scaling methods were used. The input function template was generated with scaling to the total area under the input function, because this presumable is the best scaling method [26, 28, 32]. However, it requires knowledge of the full arterial input function and therefore is not suitable for generation of population-based input functions for new subjects (without full blood-sampling).

Logan V_T_ estimated with SUV-like scaling of the population-based blood curves (PB3) and the ROI-to-cerebellum ratio showed a much weaker correlation with the reference V_T_ (Table 2, Fig. 5), suggesting that these quantitative parameters are inferior as surrogate of the true Logan V_T_.

A secondary finding of this study was the sensitivity of the reference Logan V_T_ to the TSPO gene polymorphism (Fig. 6). On average (across all ROIs), the reference Logan V_T_ was 63% larger in HAB compared to MAB subjects (range 43–93%). It was smallest in LAB subjects in all ROIs (Fig. 6). The results of previous studies have been rather inconsistent with respect to allelic sensitivity of ^18^F-GE-180 kinetics. Fan and co-workers, performing dynamic ^18^F-GE-180 PET with arterial blood sampling and metabolite correction in 10 healthy volunteers, 6 HAB and 4 MAB, found regional V_T_ to be 36–73% larger in HAB compared to MAB subjects, depending on the ROI [20]. Sridharan and co-workers, performing dynamic ^18^F-GE-180 PET with arterial blood sampling and metabolite correction in 6 patients with multiple sclerosis, 3 HAB and 3 MAB, found V_T_ in whole brain (excluding lesions) to be about 70% larger in HAB compared to MAB (estimated from fig. 5e in the publication) [50]. In contrast, Feeney and co-workers, performing dynamic ^18^F-GE-180 PET with arterial blood sampling and metabolite correction in 10 healthy volunteers, 5 HAB and 5 MAB, found no significant effect of the TSPO gene polymorphism on any regional V_T_ [21]. Studies using the SUV or scaling of regional ^18^F-GE-180 uptake to the mean uptake in a (pseudo) reference region in a late static uptake image for semi-quantitative analysis [24, 25, 51, 52] as well as studies using a reference tissue method to model dynamic ^18^F-GE-180 PET data [24] in general failed to detect a TSPO gene polymorphism effect. The present study adds further evidence for allelic sensitivity of ^18^F-GE-180 kinetics. The fact that the simplified quantification methods based on a single blood sample (i.e., PB1, PB2, ROI-to-whole-blood ratio) were also sensitive to the TSPO gene polymorphism, whereas SUV-like scaling (PB3) and the ROI-to-cerebellum ratio were not (Fig. 6, Table 2), further supports simplified quantification based on a blood sample. It also might explain the lack of a polymorphism effect in previous studies using SUV-like scaling and/or scaling to a (pseudo) reference region (i.e., these methods are not sufficiently sensitive presumably).

The lack of a genotype * ROI interaction on the Logan V_T_ in the present study suggests that the relation of V_T_ between HAB and MAB subjects is more or less constant across the brain.

Estimation of the ROI-to-whole-blood ratio might be further simplified by using a late venous rather than arterial blood sample [46], because the gradient of tracer concentration between arterial and venous blood is small due to the low single-pass extraction fraction of ^18^F-GE-180 [20, 21] (a critical discussion of this point is given in [32]). Alternatively, arterial whole-blood activity concentration might be derived from a ROI in the descending aorta in a static PET scan acquired immediately before or after the brain scan, or both, before and after. The aorta can be delineated in the low-dose CT for attenuation correction of the aorta PET with high reproducibility across observers and software tools used for delineation [53]. If there are two scans of the aorta, before and after the brain scan, a single low-dose CT can be used for attenuation correction of both. The low-dose CT of the aorta and the low-dose CT of the brain should both be performed either before the first aorta emission scan or after the second one, in order to minimize the time delay between the brain measurement and the blood measurement. The descending aorta appears more appropriate for image-based estimation of the activity concentration in arterial whole-blood than the cavum of the left ventricle, because ^18^F-GE-180 shows high uptake in the myocardium causing spill-in of activity into the cavum. Due to its high ^18^F-GE-180 uptake, the myocardium has been suggested as potential extra-cerebral tissue reference region for ^18^F-GE-180 PET [54, 55].

A further secondary finding of the present study is that the total distribution volume V_T_ of ^18^F-GE-180 was in general very low throughout the whole brain (between 0.07 and 0.20 mL/cm^3^, Fig. 6). This is in line with previous studies in healthy human subjects [20, 21, 44, 50]. Zanotti-Fregonara and co-workers performed a head-to-head comparison of ^18^F-GE-180 with the TSPO PET tracer [^11^C]PBR28 in healthy subjects and found V_T_ to be about 20 times smaller for ^18^F-GE-180 compared to [^11^C]PBR28 [44]. This most likely is explained by a low permeability-surface-area-product of brain capillaries for ^18^F-GE-180 in line with the small rate constant K_1_ for unidirectional transport of ^18^F-GE-180 from arterial blood to tissue even at normal cerebral blood flow (about 0.005 mL/min [21] to about 0.008 mL/min [20]). The low brain uptake of ^18^F-GE-180 led Zanotti-Fregonara and co-workers to question the utility of ^18^F-GE-180 for imaging neuroinflammation in humans (but not in rodent models). In response, Albert and co-workers summarized the evidence of the validity of ^18^F-GE-180 as TSPO tracer also in humans [56] (s. also [57]). Recently, Sridharan and co-workers confirmed specific binding of ^18^F-GE-180 in humans by a blocking study in patients with multiple sclerosis showing that in HAB subjects about 57% of V_T_ represent specific binding of ^18^F-GE-180 to the TSPO [50]. Simplified methods for quantitative analysis of ^18^F-GE-180 as discussed here might facilitate future studies to further evaluate ^18^F-GE-180 in humans. A systematic comparison of 13 TSPO PET and SPECT tracers including ^18^F-GE-180 is given in [58].

The following limitation of this study should be mentioned. The effect of the TSPO polymorphism on the quantitative parameters was tested by comparing them between HAB and MAB subjects independent of the group (liver-transplanted patients or healthy controls). The rationale for this was that the effect of the TSPO polymorphism on ^18^F-GE-180 binding was expected to be larger than potential effects of the patient group. This is supported by the lack of significant differences in the distribution volume of [^11^C](R)-PK11195 between cirrhotic patients with an acute episode of clinically manifest hepatic encephalopathy and healthy subjects [59]. Age, sex, and treatment (in patient group) were also not taken into account when testing for a TSPO polymorphism effect. As a consequence, we do not recommend to use the 63% increase of V_T_ in HAB compared to MAB observed in this study to correct for the TSPO polymorphism effect on ^18^F-GE-180 V_T_ for pooling data from subjects with different genotype for combined analysis. The limitations of the polymorphism analysis do not affect the primary findings of this study from the analyses of correlation between the reference V_T_ and the simplified quantitative parameters.

In conclusion, the present findings support the use of a population-based input function scaled with a single individual blood sample or the late ROI-to-whole-blood ratio for quantitative analysis of ^18^F-GE-180 PET. In the present study, an individual arterial blood sample was used for scaling. We hypothesize that the arterial blood sample can be replaced by an individual blood value derived from a late static PET scan of the descending aorta without compromising the validity of simplified quantification as a surrogate for Logan V_T_.

## Electronic supplementary material

ESM 1 (DOCX 2834 kb)

## Data Availability

All data generated and analyzed during this study, including the input function templates, are available for readers on request.
