# Supplementary material for: Reliable quantification of 18F-GE-180 PET neuroinflammation studies using an individually scaled population-based input function or late tissue-to-blood ratio
Source: Eur J Nucl Med Mol Imaging. 2020 Apr 23;47(12):2887–900. doi: 10.1007/s00259-020-04810-1 (PMC7651670; doi:10.1007/s00259-020-04810-1)
Supplement: Supplementary file 1 — (DOCX 2834 kb) [file 259_2020_4810_MOESM1_ESM.docx]

**Reliable quantification of ^18^F-GE-180 PET neuroinflammation studies using an individually scaled population-based input function or late tissue-to-blood ratio**

**Supplementary**

**Brain regions of interest**

Version AAL3 (released 27 August 2019) of the automatic anatomic labeling atlas [1, 2] was used to define the following bilateral regions of interest (ROI) in the anatomical space of the Montreal Neurological Institute (MNI): frontal (union of AAL3 ROIs 1-32), parietal (63-70), temporal without mesial temporal (85-94), occipital (47-58), cerebellum (95-112), insula (33-34), cingulate (35-40), mesial temporal (41-46), precuneus (71-72), striatum (75-78), and thalamus (121-150). A ROI of the superior longitudinal fasciculus was taken from the white matter tractography atlas of the Johns Hopkins University [3], because the AAL3 atlas does not provide white matter ROIs. In order to minimize spill-in from large vessels, a large vessels ROI was created by first averaging the globally scaled PET image frame in MNI space with highest tracer concentration in large vessels over all subjects (frame 2 in most subjects, corresponding to 15-30s post injection). The large vessels ROI was obtained by thresholding the resulting mean image at 20% of its maximum. Voxels included in the large vessel ROI were removed from the tissue ROIs. The resulting tissue ROIs are shown in figure S1 together with the large vessels ROI.

**Correction for delay and dispersion**

An image-derived TAC of arterial whole-blood was obtained as follows. A dynamic series of twenty PET images each of 3 s duration was reconstructed from the first minute of the PET list mode emission data. In order to eliminate potential reconstruction artefacts at the axial edges of the field-of-view, the first three transaxial planes and the last three transaxial planes were removed from each image. Then each image was filtered with an isotropic 3-dimensional Gaussian kernel of 6 mm full-width-at-half-maximum (FWHM). The mean activity concentration of the 6113 hottest voxels (corresponding to 30 ml) was determined separately for each image frame and plotted as a function of the frame number (Figure S2). The frame at which the activity started to increase towards the peak activity was determined visually (frame 6 corresponding to 15-18 s post injection in most subjects, Figure S2). A binary mask for arterial whole-blood was defined by the 6113 hottest voxels in this frame (Figure S3). The resulting mask was checked visually in each subject. In addition to the carotid arteries, the mask included smaller arteries in most subjects (e.g., communicans posterior, cerebri posterior, basilaris and superior cerebelli arteries, Figure S3). The image-derived whole-blood TAC was obtained by applying the binary whole-blood mask to each frame (mean activity concentration over all voxels in the mask).

Delay and dispersion of the whole-blood TAC measured with the automatic blood sampler relative to the image-derived whole-blood TAC in the brain were estimated and then correct for as described in [4]. More precisely, dispersion was modeled by convolution with the dispersion kernel $\left( 1/\tau\right)e^{-t/\tau}$ [5], where $\tau$ denotes the time constant of dispersion. Delay was modeled by shifting the time scale of the measured whole-blood TAC by the time delay $\Delta$, that is

$\left( 1/\tau\right)e^{-t/\tau}*a \left( image derived whole blood TAC \right)= C_{m}\left( t+\Delta\right)$, (S1)

where “*” denotes convolution, *a* is a scale factor to adjust the amplitude of the image-derived whole-blood TAC (to account for partial volume effects), and $C_{m}$ is the whole-blood TAC measured by the blood sampler. The parameters *a,* $\tau$*,* and $\Delta$ were determined by minimizing the sum of squared residuals using the MATLAB function fminsearch.

The whole-blood TAC was corrected for the estimated delay and dispersion using the formula [4]

$C_{corr}\left( t \right)= C_{m}\left( t+\Delta\right)+\tau\dot{C}_{m}\left( t+\Delta\right)$, (S2)

where $C_{corr}$ is the corrected whole-blood TAC and $\dot{C}_{m}$ is the time derivative of the measured whole-blood TAC $C_{m}$. In order to reduce amplification of noise by estimating $\dot{C}_{m}$ from the noisy $C_{m}$, $C_{m}$ was fitted by the model

$C_{m}\left( t \right)=\left\{ \begin{matrix} f_{1}\left( t \right)= & 0, & t\leq t_{0} \\ f_{2}\left( t \right)= & \left( t-t_{0} \right)\left[ a_{1}e^{-\mu_{1}\left( t-t_{0} \right)}+a_{2}e^{-\mu_{2}\left( t-t_{0} \right)} \right], & t_{0}\leq t<t_{1} \\ \begin{matrix} f_{3}\left( t \right)= \\ f_{4}\left( t \right)= \end{matrix} & \begin{matrix} a_{3}e^{-\mu_{3}\left( t-t_{1} \right)}+\left[ f_{2}\left( t_{1} \right){-a}_{3} \right]e^{-\mu_{4}\left( t-t_{1} \right)}, \\ a_{4}e^{-\mu_{5}\left( t-t_{2} \right)}+\left[ f_{3}\left( t_{2} \right){-a}_{4} \right]e^{-\mu_{6}\left( t-t_{2} \right)}, \end{matrix} & \begin{matrix} t_{1}\leq t<t_{2} \\ t\geq t_{2} \end{matrix} \end{matrix} \right.$ (S3)

The quality of the fit was checked visually and was found to be adequate in all cases (Figure S4). An estimate of $\dot{C}_{m}$ was obtained using difference quotients computed from the model fit. The resulting curve was filtered with a 1-dimensional Gaussian kernel with 0.25 s FWHM in order to avoid artefacts at the separation points $t_{0}, t_{1}, t_{2}$ of the piecewise model (S3). The model was only used to estimate $\dot{C}_{m}$. The first term on the right hand side of formula (S2) was obtained from the measured (noisy) $C_{m}$. A representative example of the corrected whole-blood TAC is shown in Figure S5.

**References**

1. Rolls ET, Joliot M, Tzourio-Mazoyer N. Implementation of a new parcellation of the orbitofrontal cortex in the automated anatomical labeling atlas. Neuroimage. 2015;122:1-5. doi:10.1016/j.neuroimage.2015.07.075.

2. Tzourio-Mazoyer N, Landeau B, Papathanassiou D, Crivello F, Etard O, Delcroix N, et al. Automated anatomical labeling of activations in SPM using a macroscopic anatomical parcellation of the MNI MRI single-subject brain. Neuroimage. 2002;15:273-89. doi:10.1006/nimg.2001.0978.

3. Hua K, Zhang JY, Wakana S, Jiang HY, Li X, Reich DS, et al. Tract probability maps in stereotaxic spaces: Analyses of white matter anatomy and tract-specific quantification. Neuroimage. 2008;39:336-47. doi:10.1016/j.neuroimage.2007.07.053.

4. van den Hoff J, Burchert W, Muller-Schauenburg W, Meyer GJ, Hundeshagen H. Accurate local blood flow measurements with dynamic PET: fast determination of input function delay and dispersion by multilinear minimization. J Nucl Med. 1993;34:1770-7.

5. Iida H, Kanno I, Miura S, Murakami M, Takahashi K, Uemura K. Error analysis of a quantitative cerebral blood flow measurement using H2(15)O autoradiography and positron emission tomography, with respect to the dispersion of the input function. J Cereb Blood Flow Metab. 1986;6:536-45. doi:10.1038/jcbfm.1986.99.

**Supplementary figures**

**
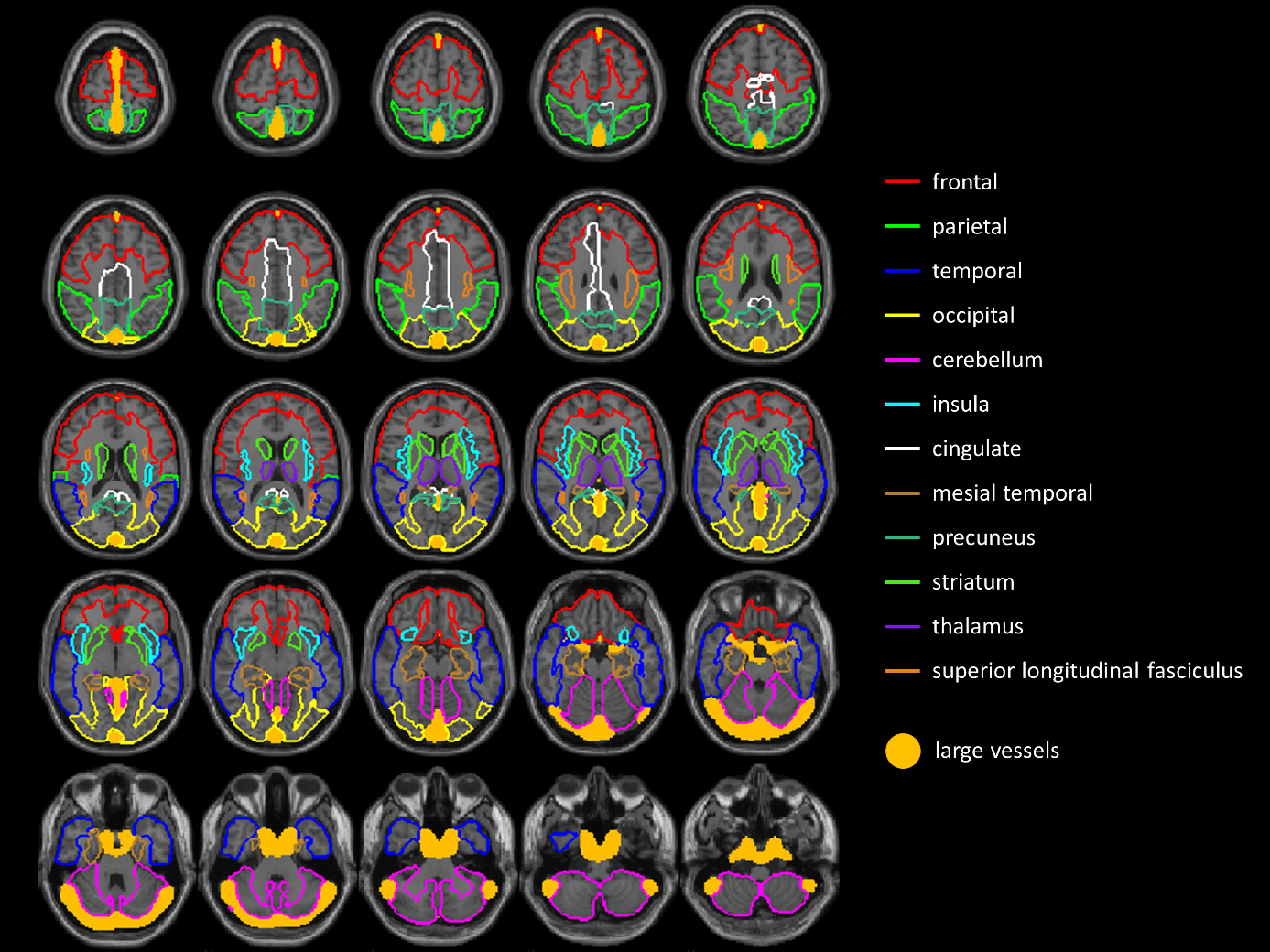
**

**Fig. S1** Regions of interest in MNI space


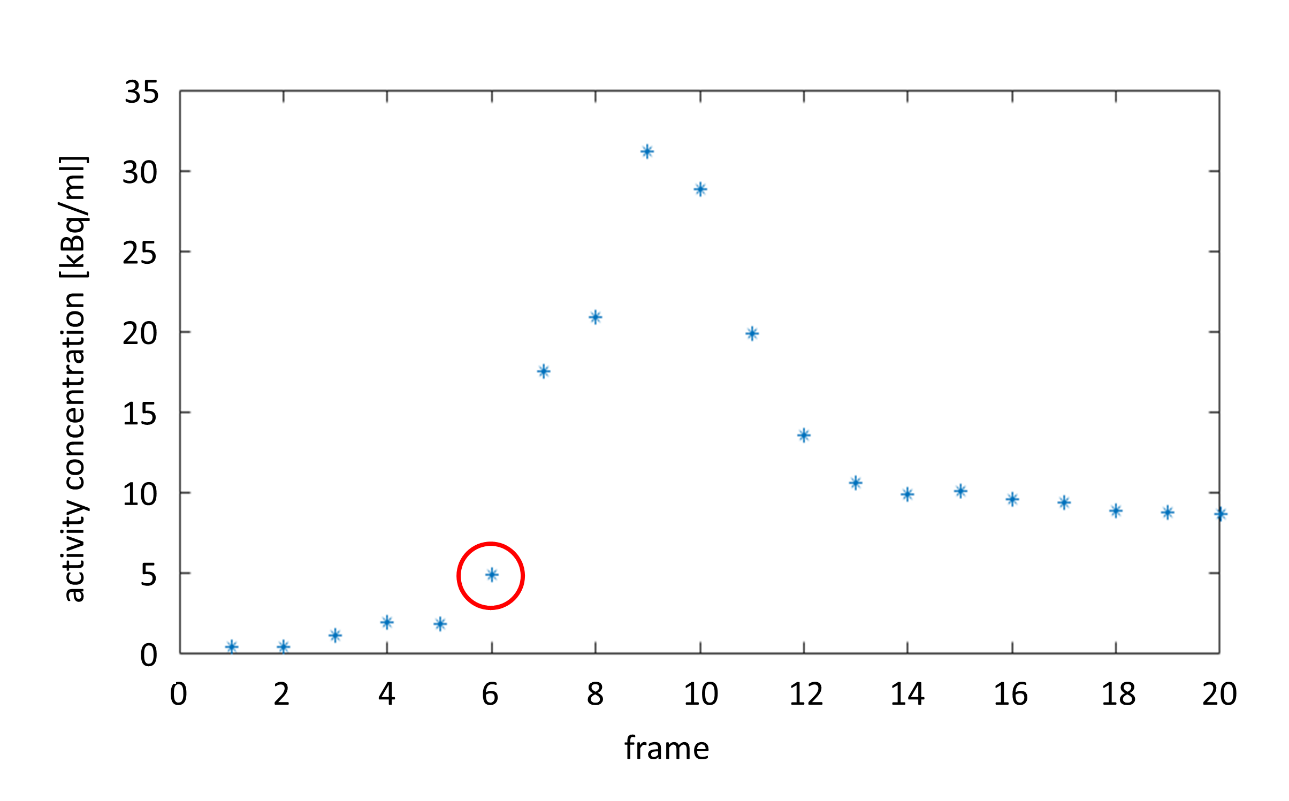


**Fig. S2** Mean activity concentration of the 6113 hottest voxels (corresponding to 30 ml) in 20 consecutive PET images of 3 s duration reconstructed from the first minute of the PET list mode emission data of a representative subject. The hottest voxels were determined separately for each frame. The frame at which the activity started to increase towards the peak activity was determined visually (here frame 6, red circle)


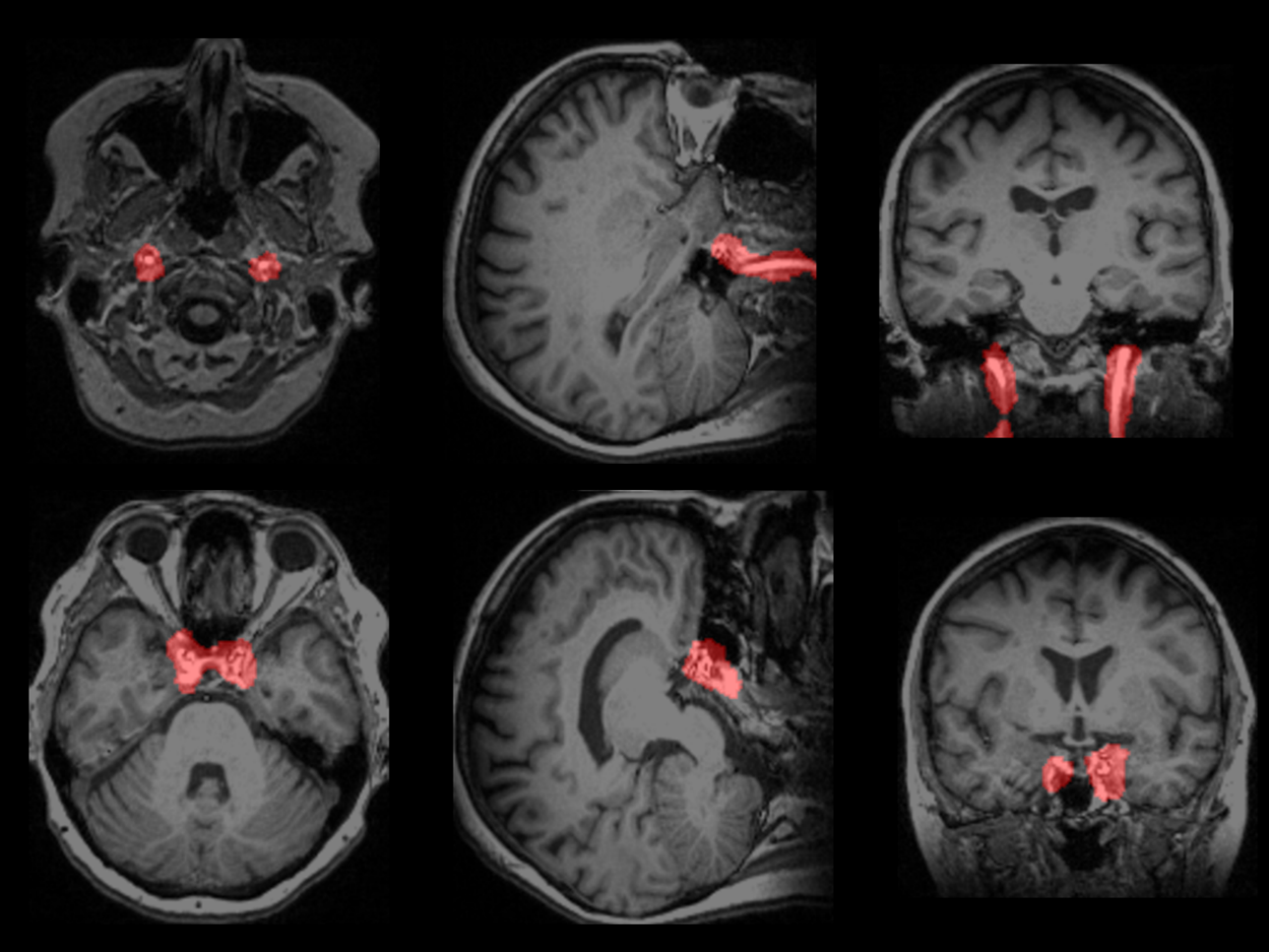


**Fig. S3** Binary arterial whole-blood mask in a representative subject. The mask is overlaid to the subject’s T1w-MRI


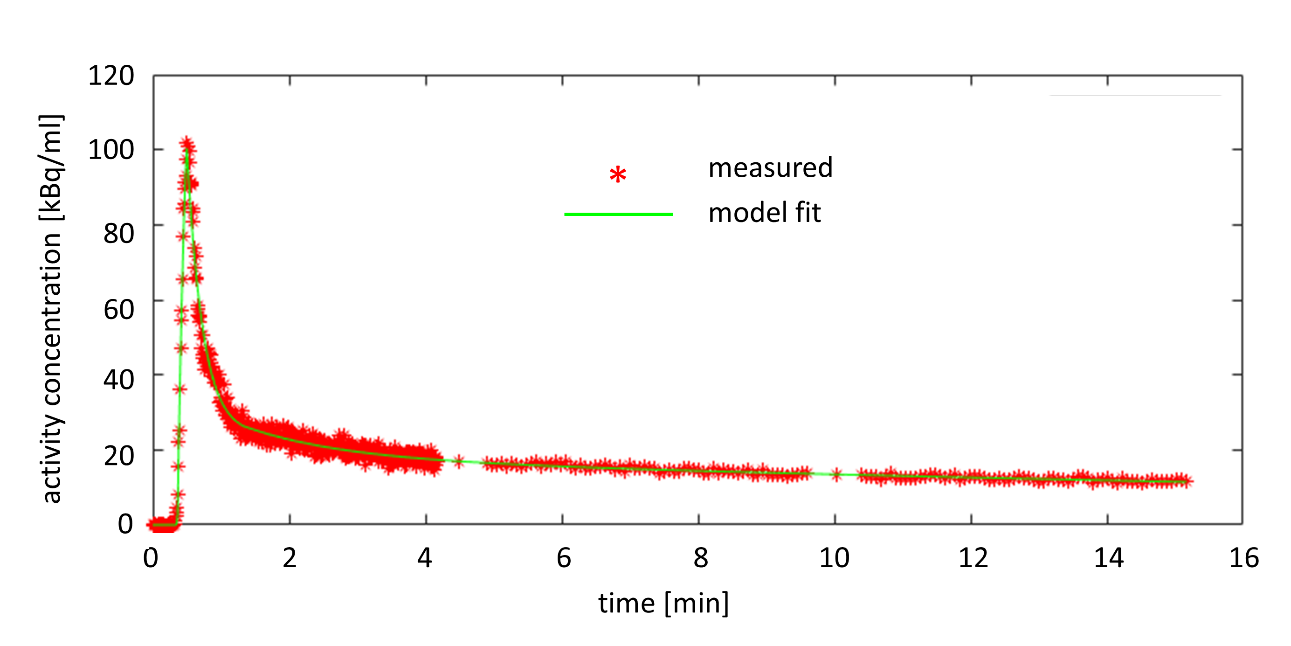


**Fig. S4** Model fit of the whole-blood TAC measured with the automatic blood sampler


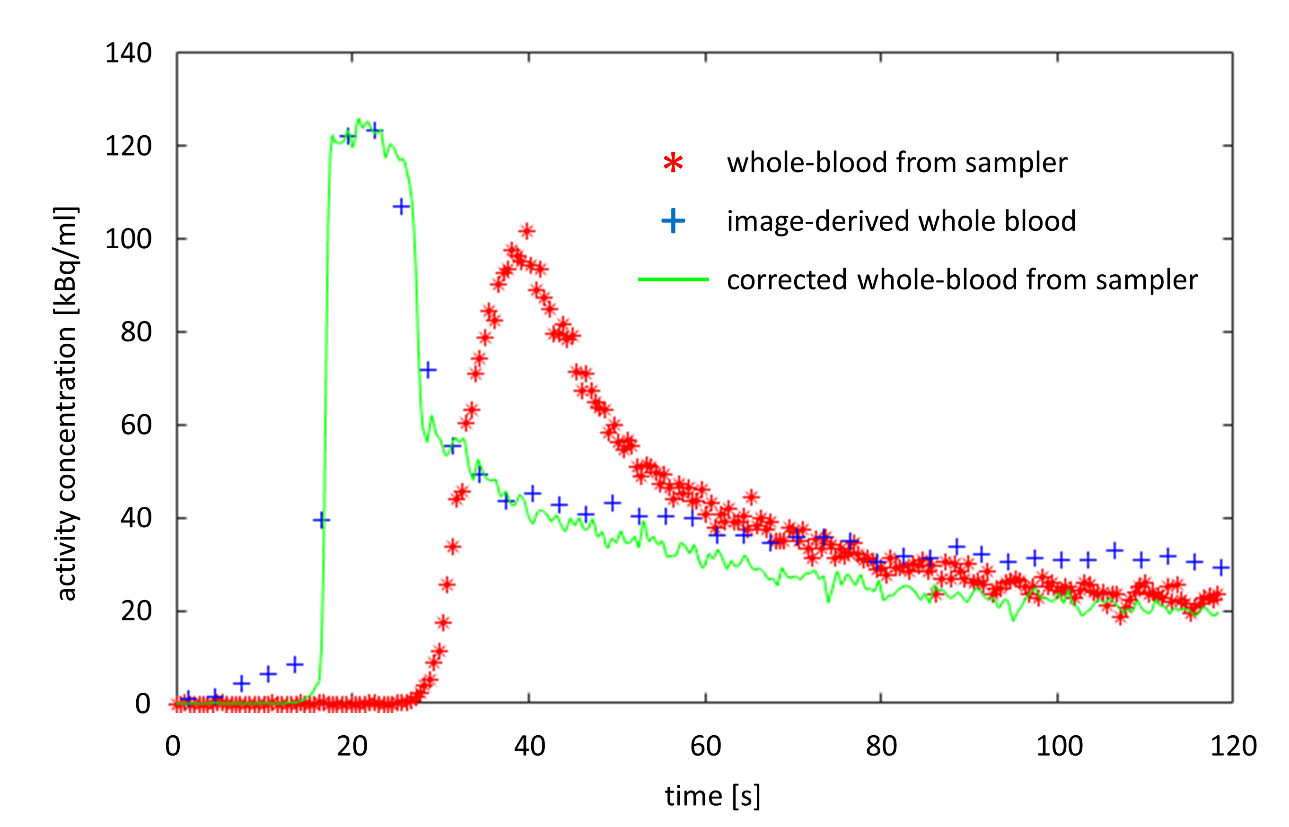


**Fig. S5** Representative whole-blood TAC measured with the automatic blood sampler corrected for delay and dispersion based on an image-derived whole-blood TAC. Delay and dispersion time constant were estimated to $\Delta=$12.3 s and $\tau=$5.7 s in this case
